# Zinc oxide nanorod field effect transistor for long-time cellular force measurement

**DOI:** 10.1038/srep43661

**Published:** 2017-03-08

**Authors:** Xianli Zong, Rong Zhu

**Affiliations:** 1 State Key Laboratory of Precision Measurement Technology and Instruments, Department of Precision Instrument, Tsinghua University, Beijing 100084, China

## Abstract

Mechanical forces generated by cells are known to influence a vast range of cellular functions ranging from receptor signaling and transcription to differentiation and proliferation. We report a novel measurement approach using zinc oxide nanorods as a peeping transducer to monitor dynamic mechanical behavior of cellular traction on surrounding substrate. We develop a ZnO nanorod field effect transistor (FET) as an ultrasensitive force sensor to realize long-time, unstained, and *in-situ* detection of cell cycle phases, including attachment, spread, and mitosis. Excellent biocompatibility and ultra-sensitivity of the biomechanical measurement is ensured by coating a parylene film on the FET sensor as a concealment, which provides complete electronic isolation between the sensor and cell. With unique features of ultra-sensitivity, label-free, easy handling, and good biocompatibility, the force sensor allows feasible for tracking cellular dynamics in physiological contexts and understanding their contribution to biological processes.

Studies on mechanics of cells have rapidly evolved during the past decade with significant implications for biotechnology and human health^1^. Cellular force measurement can be divided into two categories: active methods and passive methods. Active methods utilizing atomic force microscopy (AFM)^2^ or optical tweezers^3^ exert external forces onto cells to induce cellular signaling or to characterize mechanical properties (such as stiffness). These approaches provide high sensitivity and resolution for cellular force measurement. However, they rely heavily on external physical equipment and scientific facilities^4^. Passive methods measure forces exerted by cells interacting with a surrounding substrate which reveal cellular functions, such as adhesion, spread, division, migration, and necrosis^5^. Researchers have taken great efforts to actualize cellular force measurements by using deformable cellular traction force microscopy (TFM)^6^, micro-fabricated platforms^7^, micropillar^8^, etc. TFM involves tracking deformations of synthetic elastic polymer substrates resulted from exertion of cellular force. This method allows cellular forces to be mapped with subcellular resolution. However, the calculations are complex, nuanced, and difficult to validate, which has been a significant challenge for further improvement^5^,^6^. Micro-fabricated platforms have been developed to measure cellular tractions directly in idealized mechanical environments. Cellular forces are converted to electrical signals by deformable silicon elements on chip^7^. Microfabricated arrays of elastomeric micropillars have been developed to manipulate and measure mechanical interactions between cells and underlying substrates^8^. The displacement of each pillar can be tracked and the applied force can be calculated by fluorescent labeling. However, the measurement usually relies on confocal microscopy.

Electrical measurements are real-time, *in-situ*, and label-free tools for cell study. It is easy to realize device miniaturized. Conventional electrical measurements such as cell impedance measurement, have been employed to study cytotoxic effects of various substances^9^, cell response to drug treatment^10^, cell adhesion and spread^11^,^12^, cell death^13^, cell cycle^14^, cell migration^15^ and etc. However, electrical measurements are easily interfered by spurious signals from such as medium, double-layer capacitance on electrode-electrolyte interface, and parasitic capacitances, which may greatly damage cell measurement, especially for single cell detection. Furthermore, electrolysis on electrode surface causes a pH change of cell medium that suppresses viability of cells, even leads them to death.

Here we propose an alternative electrical measurement approach to monitor dynamic mechanical behavior of cellular traction for a long time. Zinc oxide nanorods (ZnO NRs) are utilized as force transducer. Semiconducting ZnO nanorods are one-dimensional nano-structures that have been used in many nano-devices, such as transistors^16^,^17^, resonators^18^, and nano-sensors^19^,^20^,^21^. They are pure, structurally uniform, single crystalline, and most are free from dislocation. Many unique properties of ZnO have been demonstrated, such as piezoelectricity. Here, a ZnO nanorod field effect transistor (FET) is proposed. ZnO nanorods are synthesized at the opposite ends of two micro electrodes and cross connected between the electrodes by using an electric-field assisted hydrothermal growth method^22^ to form a FET. Forces exerted by cells onto underlying ZnO nanorods are transduced into Schottky barrier change at semiconductor/metal interface for piezoelectricity of ZnO, which is detected by using a frequency mixing effect of semiconducting nanorods and a lock-in amplifier. The ZnO nanorod FET force sensor is concealed by depositing a parylene film on the sensor to isolate electrical measurement from cell environment and thus ensures an excellent biocompatibility for long time measurement. In addition, the sensor has good anti-jamming capability due to the parylene film isolation and possesses high sensitivity by using the frequency mixing of FET and lock-in detection.

The nanorod-based FET force sensor is employed to measure cellular dynamic activities continuously for 48 hours and successfully demonstrates typical phases in cell cycles, such as cell adhesion, spread, interphase, and proliferation. With impressive merits of high sensibility, label-free, easy handling, and good biocompatibility, the cell force sensor can be applied for measuring cellular forces in a physiological context and understanding their contribution to biological processes in drug screening, cell differentiation, cancer cell detection, diseases diagnosing, wound healing, and etc^23^,^24^.

## Results

### Configuration of force sensor and measuring system

**Figure 1:**
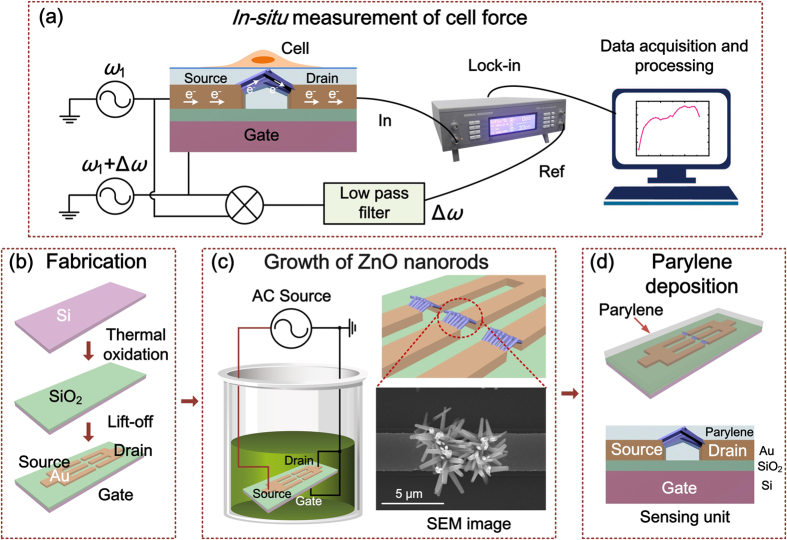
Schematic view of the force sensor and measuring system, as well as fabrication process of the sensor. (**a**) Setup of force sensor and measuring system. AC voltage with a frequency of *ω*_1_ is applied on source electrode and another AC voltage with a frequency of *ω*_1_ + ∆*ω* is applied on gate electrode. The current at ∆*ω* through the nanorods is detected by a lock-in amplifier. (**b**) Fabrication process of the FET micro-chip. (**c**) Growth of ZnO nanorods between source and drain. (**d**) Parylene depositing process.

Configuration of ZnO nanorod FET force sensor is shown in Fig. 1a. The sensor is structured with source, drain and gate electrodes, as well as semiconducting ZnO nanorods serving as conductive channel of the FET. The force sensor incorporating with a lock-in amplifier constructs a measuring system.

The fabrication of the force sensor starts with preparation of a FET microchip comprising three electrodes — source, drain and gate, as shown in Fig. 1b. Afterwards, ZnO nanorods are synthesized site-selectively at the opposite ends of the Au source and drain electrodes, and cross connected between two electrodes by using an electric-field assisted wet chemical method (Fig. 1c)^22^. The growth of ZnO nanorods can be controlled by applying certain electric field on the microchip. The fabricated ZnO nanorods is about 1~2 μm in length and about 300~500 nm in diameter. The EDX results of ZnO nanorods prove the atomic ratio of zinc to oxygen is near the stoichiometric composition (1:1). TEM images of ZnO nanorods, SAED pattern and HRTEM examination indicate the single crystal structure of ZnO nanorods and their <0001> growth direction^22^.

After the fabrication of ZnO NRs, a 1.8 μm thick parylene film is deposited on the sensor as an encapsulation (Fig. 1d) to ensure good biocompatibility and electronic isolation. The thickness of the parylene film is optimized according to sensor stability and sensitivity. The fabricated sensor is connected with a mixer circuit. An alternating current (AC) sine-wave voltage with a frequency of *ω*_1_ is applied to the source, and another AC voltage with a frequency of *ω*_1_ + Δ*ω* is applied to the gate. The difference frequency current at Δ*ω* through the ZnO nanorods from source to drain is detected by the lock-in amplifier.

### Sensing Mechanism of the force sensor

**Figure 2:**
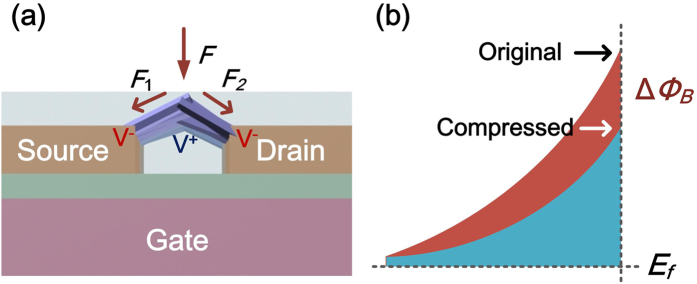
Mechanism of force transducing principle by piezotronics. (**a**) Sketch of external forces applied on ZnO nanorods. (**b**) Corresponding energy band diagrams at ZnO/Au interface on two states.

Sensing Mechanism of the force sensor is shown in Fig. 2. External forces exerted onto ZnO nanorods generate compressions on nanorods, which are converted to Schottky barrier changes at ZnO/electrode interfaces due to piezoelectricity of ZnO nanorods^25^. According to the metal-semiconductor contact theory, the *I-V* characteristics of the ZnO/electrode contact in a forward bias is given by ref. 26


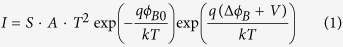


where *S* is the contact area, *A* is the effective Richardson constant, *k* is Boltzmann constant, *T* is the temperature, *q* is the electron charge, 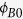 is Schottky barrier height at null force, 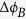 is the force-induced lowering of Schottky barrier. The *I-V* characteristics of ZnO nanorod FET chip demonstrate symmetric Schottky contacts at two ends of nanorods (Figure S1).

When the ZnO nanorod is compressed, piezoelectric potential is generated along its length (c-axis of the nanorod) as shown in Fig. 2a. The generated potential decreases the Schottky barrier (Fig. 2b) in the form of ref. 27.


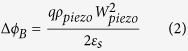


where 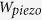 is the width of the polarization layer on the interface, 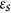 is the permittivity of ZnO, and 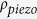 is the density of polarization charges (in units of electron charge) that is proportional to the strain *ε* along c-axis of the nanorod. Consequently, the Schottky barrier change, 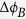, is proportional to strain *ε*. When the nanorod is compressed, the strain-induced negative charge on the ZnO/Au interface increases the current under a certain applied voltage. Equations (1) and (2) indicate that the effective conductance *G* (from source to drain) of the FET sensor is definitely relevant to the external forces that compress ZnO nanorods in the FET.

**Figure 3:**
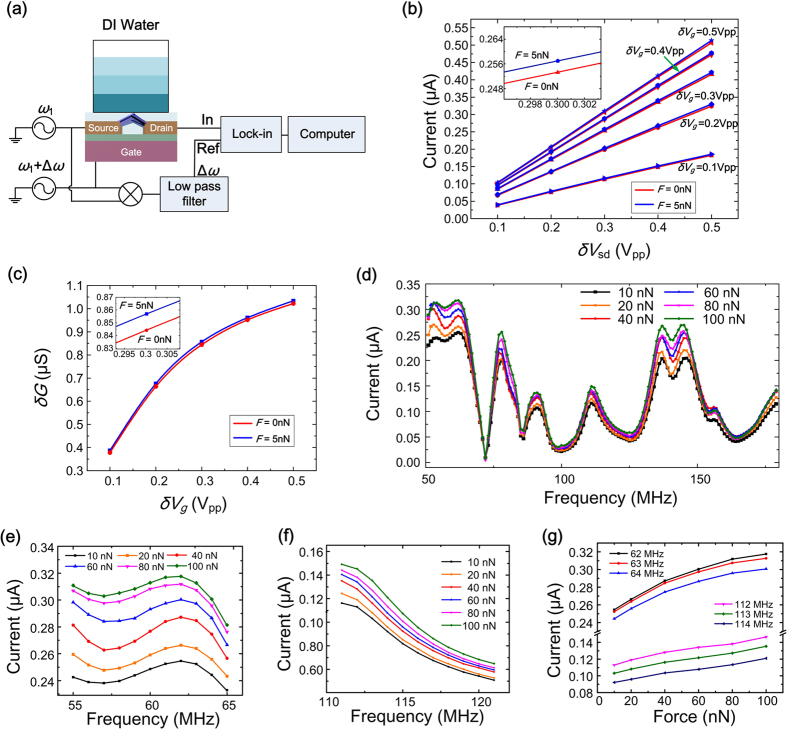
Calibration of FET force sensor. (**a**) Sketch of calibration process. (**b**) Detected lock-in currents (*ω*_1_ = 62 MHz, ∆*ω* = 30 kHz) versus peak-to-peak values of *δV*_*sd*_ and *δV*_*g*_ under a force of 5 nN and 0 nN respectively. Inset is the enlarged image at *δV*_*g*_ = 0.3 V_pp_ and *δV*_*sd*_ = 0.3 V_pp_. (**c**) Calculated *δG* versus *δV*_*g*_ under different forces. (**d**) Lock-in currents (∆*ω* = 30 kHz) of the sensor by a frequency sweep of *ω*_1_ ranging from 50 MHz to 180 MHz at a frequency step of 1 MHz under a force increasing from 10 nN to 100 nN. (**e**,**f**) Enlarged images of (**d**) in the frequency range of 55~60 MHz and 111~121 MHz respectively. (**g**) Detected lock-in currents versus the exerted force at different frequencies.

To detect the conductance change of the FET induced by the external force, a frequency mixing scheme is utilized to achieve high sensitivity of detection. As shown in Fig. 1a, an AC voltage *V*_*sd*_ with a frequency of *ω*_1_ is applied on source with another AC voltage *V*_*g*_ with a frequency of *ω*_1_ + ∆*ω* applied on gate. The conductance change of the FET through semiconducting nanorods can be modulated by the gate voltage given by ref. 28.


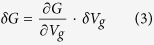


where 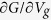 is the conductance change versus gate voltage, which is altered by Schottky barrier change 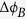 correlated with external force exertion (the evidence is shown in Fig. 3b,c). The current change 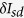 through semiconducting nanorods is given by the product of the source voltage *V*_*sd*_ and the conductance change 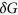. Therein, the difference frequency current at ∆*ω* detected by the lock-in amplifier is given by.


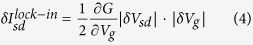


where 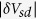 and 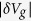 are amplitudes of AC voltages *δV*_*sd*_ and *δV*_*g*_ respectively. The above frequency mixing detection strengthens the inhibition capability of the sensor against interferences, which means only the external force exerted onto ZnO nanorods resulting in the change of conductance can be detected. Moreover, the frequency *ω*_1_ can be modulated to megahertz, much higher than ∆*ω* at kilohertz, which further enhances the measurement accuracy of the lock-in detection.

In measurement, the force sensor transduces the cellular force into conductance change of the FET, which is further converted into the difference frequency current through ZnO nanorods and detected by a lock-in amplifier.

### Calibration of the force sensor

To characterize the force sensor, calibration experiment is conducted by using a controllable hydraulic pressure to exert reference force on the sensor, which is adjusted by altering the height of deionized water above the force sensor, as shown in Fig. 3a.

Figure 3b and Figure S2 show the detected lock-in currents (*ω*_1_ = 62 MHz, ∆*ω* = 30 kHz) of the force sensor versus the peak-to-peak values of *δV*_*sd*_ and *δV*_*g*_ under a force of 0 nN and 5 nN respectively. Results show that with the increase of *δV*_*sd*_ from 0.1 Vpp to 0.5 Vpp, the lock-in current increases proportionally. While with the increase of *δV*_*g*_ from 0.1 Vpp to 0.5 Vpp, the lock-in current grows with a slowing speed. Inset image in Fig. 3b shows the current responses with different forces under *δV*_*g*_ = 0.3 Vpp and *δV*_*sd*_ = 0.3 Vpp. Figure 3c is the calculated *δG* versus *δV*_*g*_ under different forces to validate the supposition that the conductance of the FET is correlated with the external force. The above experiment results demonstrate the correlation between the lock-in current of the FET sensor and the external force exerted on the sensor.

Results of the sensor calibration with the exerted force increasing from 10 nN to 100 nN are shown in Fig. 3d. The frequency spectrum reveals the frequency response of the ZnO nanorod FET sensor versus the frequency *ω*_1_ sweeping from 50 MHz to 180 MHz, illustrating the intrinsic property of the whole measurement system dominated by the inductances, capacitances and resistances of the system. Figure 3e,f are enlarged images of Fig. 3d with sweep frequency in a range of 55~65 MHz and 111~121 MHz respectively. Figure 3g shows the detected lock-in currents versus the exerted force at different specific frequencies, which indicates that the lock-in current of the sensor increases with the exerted force. To obtain good linearity in cellular force detection, we select 110~120 MHz as a sweep frequency range for the sensor. In this range, the sensitivity of the force sensor reaches about 0.4 nA/nN. The standard error of the sensor output is about 0.1 nA, which corresponds to a resolution of about 0.25 nN.

### Long time *in-situ* measurement of Hela cells

**Figure 4:**
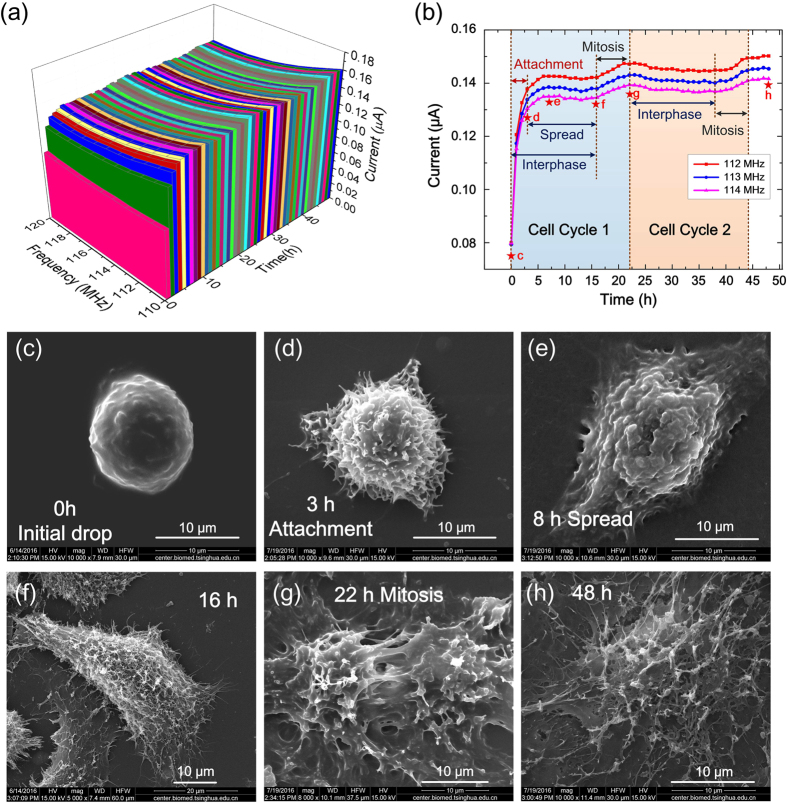
Long time *in-situ* measurement of living Hela cell. (**a**) The lock-in current of the sensor in a frequency sweep ranging from 110 MHz to 120 MHz detected continuously for 48 hours. (**b**) The detected lock-in current changing over time at certain frequency, where different phases of cell cycle are indicated. (**c**–**h**) SEM images of living Hela cells rapidly frozen at different phases of cell cycles: (**c**) Initial drop, (**d**) Attachment, (**e**) Spread, (**f**) 16 h, (**g**) Mitosis in the first cycle and (**h**) Interphase in the third cycle. The phases in (**c**–**h**) correspond to the time points marked as stars in (**b**) respectively.

To verify the long-time stability, the sensor immerging in cell culture is tested continuously for 14 hours. The testing result (Figure S3) shows excellent long-time stability. Based on this, a real-time and *in-situ* measurement on living Hela cell using the force sensor is conducted continuously for 48 hours. As mentioned above, a sweep frequency range of 110~120 MHz and a difference frequency of 30 kHz are selected for the measurement of Hela cells. Results are shown in Fig. 4a,b.

The curves in Fig. 4(b) reveal cellular dynamic behaviors in more than two cell cycles, where typical phases are observed including cell attachment, cell spread and cell division. Figure 4c–h show SEM images of the contemporaneous Hela cell rapidly frozen at different stages corresponding to the time point marked in Fig. 4b. The experimental results indicate that the cell in suspension (Fig. 4c) attaches and adheres onto the substrate in 0~3 hours, called attachment (Fig. 4d). In this period, the cell adhesive force increases rapidly, which can be identified from the significant increase of the sensor current shown in Fig. 4b. Then the cell spreads out during 3~8 hours with a slow increase of cellular force, exhibiting a gradual increase of the sensor current, (Fig. 4e). Afterwards, in 8~16 hours, cellular force decreases slightly and the sensor current exhibits relatively stable (Fig. 4f). Finally, the cell enters into the mitosis phase in 16~22 hours, in which the sensor current rises obviously as a result of cell division (Fig. 4g). In this period, the tensile force between two divisional cells becomes larger which increases the extrusion force applied on ZnO nanorods. As a result, the measured current increases. After a completion of one cycle, the cell enters into second cycle in 22~44 hours, successively passing through cell interphase and cell division that can be obviously seen in Fig. 4b,h shows the cell at interphase in the third cell cycle.

Based on above measurements, it can be deduced that the cell adhesion traction force is about 120~150 nN, the cellular force gains an increase of about 10~15 nN in spreading, and additional increase of about 15 nN in cell division. Reproducibility of measurement using different force sensor is shown in Figure S4, which demonstrates similar response behavior in cellular adhesion process.

## Discussion

In this paper, a FET-based force transistor using semiconducting ZnO nanorods is implemented to investigate long-time dynamic interaction between cell and substrate. Piezoelectricity of ZnO nanorods transduces cellular forces into Schottky barrier changes at ZnO/metal interfaces and thus modulates drain current of the FET detected by a lock-in amplifier. Semiconducting ZnO nanorod based FET serving as a frequency mixer ensures excellent anti-jamming capability for the force detection. A parylene film concealing the force sensor ensures good biocompatibility and electronic isolation. Sensitivity of the force sensor reaches about 0.4 nA/nN and the resolution is about 0.25 nN. Real-time, label-free, *in-situ* and non-intrusive detection of cell cycles continuously monitored for 48 hours is realized using the force sensor and comprehensively analyzed by comparing the sensor signals with SEM images of cell morphologies at different phases, which reveals a high relevance between the sensor output and cell physiological contexts.

With the advantages of low cost, easy handling, high sensitivity, and biocompatibility, this novel force sensing approach has great potential for measuring cellular mechanical properties and understanding their contributions to biological processes in drug screening, cell differentiation, cancer cell detection, diseases diagnosing, angiogenesis, inflammation, wound healing, and etc.

## Methods

### Fabrication process

The fabrication of the force sensor starts with preparation of a FET microchip comprising three electrodes of source, drain and gate, as shown in Fig. 1b. Afterwards, ZnO nanorods are synthesized site-selectively at the opposite ends of the Au source and drain electrodes, and cross connected between two electrodes by using an electric-field assisted wet chemical method (Fig. 1c). An AC sine-wave voltage (1 MHz, 7.0 Vpp generated by Tektronix Arbitrary/Function Generator AFG 3252) is applied on the source electrode while the drain and gate electrodes are grounded. After 5 hours of nanorod growth process, the microchip is picked up from the solution, rinsed with deionized water using ultrasonic cleaning and dried in air.

The growth of ZnO nanorods can be controlled by applying certain electric field on the microchip and detailed discussions can be found in our previous paper^22^. The fabricated ZnO nanorods is about 1~2 μm in length and about 300~500 nm in diameter. EDX results of ZnO nanorods prove the atomic ratio of zinc to oxygen is near the stoichiometric composition (1:1). TEM images of ZnO nanorods, SAED pattern and HRTEM examination indicate the single crystal structure of ZnO nanorods and their <0001> growth direction. The structural properties of ZnO nanorods are characterized using SEM (JSM-6460LV)^22^.

After the fabrication of ZnO nanorods, a 1.8 μm thick parylene film is deposited on the sensor as a concealment (Fig. 1d) to ensure good biocompatibility and electronic isolation using PDS LABCOTER deposition system. The film thickness is measured by using a step profiler Veeco Dektak 150. The thickness of the parylene film is optimized according to sensor stability and sensitivity. The fabricated sensor is connected into a frequency mixer circuit, where an alternating current (AC) sine-wave voltage with a frequency of *ω*_1_ is applied to the source, and another AC voltage with a frequency of *ω*_1_ + Δ*ω* is applied to the gate. The difference frequency current at Δ*ω* through the ZnO nanorods from source to drain is extracted by the lock-in amplifier.

### Calibration of the force sensor

Calibration experiment is conducted by adjusting the height of the deionized water above the force sensor, as shown in Fig. 3a. The external force exerted on ZnO nanorods is inferred by


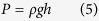



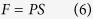


where *P* is the hydraulic pressure, *ρ* is the density of deionized water, *g* is gravitational acceleration, and *h* is the height of the water. *F* is the force exerted on ZnO nanorods and *S* is an effective area of nanorods.

According to equations (5) and (6), the height of water column for 10 nN is 5.7mm (*ρ* = 1.0 g/cm^3^, *g* = 9.8 N/Kg, S = 180 μm^2^). The height of water column increases proportionally to the force applied as 0.57 mm/nN.

Sine wave voltage with a frequency of *ω*_1_ is applied on source electrode and another sine wave voltage with a frequency of *ω*_1_ + ∆ω is applied on gate electrode. Sine wave signals are generated by using Tektronix Arbitrary/Function Generator AFG 3252. The difference frequency current at ∆*ω* frequency is detected by using a lock-in amplifier (Signal Recovery 7265 DSP).

The frequency sweep is generated by the process: the frequency of the input signal is incremented in discrete steps from the start to the stop frequency, with a dwell time at each point to allow the system to stabilize. This process can be visualized as a stepwise climbing. The time resolution *T*_*r*_ of the force sensing is determined by the frequency step *f*_*step*_, the dwell time *T*_*dwell*_, and the sweep range *f*_*b*_.


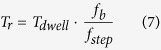


In cellular force detections, we set the frequency step as 1 MHz, the dwell time as 0.5 s, and the sweep range is 10 MHz, thereby the time resolution of the force sensing is deduced to be 5 s, which is sufficient for cellular dynamic detection.

### Cell Seeding and cell force measurement

Hela cells are cultured as a monolayer in a 60 mm Petri dish containing Dulbecco’s Modified Eagle Medium (DMEM) supplemented with 10% fetal bovine serum at 37 °C and 5% CO_2_ atmosphere. The cells are harvested from the Petri dish at the log phase of growth by 0.25% trypsin/EDTA, and then are re-suspended in the culture medium at a concentration of 2 × 10^5^ cells mL^−1^. The Hela cells suspension is poured into a pool of the micro-chip. The polypropylene (PP) culture pool is adhered on the microchip using room temperature vulcanizing (RTV) silicone rubber (WD 6705 purchased from KANGDA NEW MATERIALS CO.) to form a cell culture pool in advance.

The real-time *in-situ* cell force measurement is conducted by incubating living Hela cell at 37 °C in a 5% CO_2_ atmosphere in a cell culture incubator. The measurement is performed by applying two AC sine-wave voltages generated by Tektronix Arbitrary/Function Generator AFG 3252 with the same magnitude of 0.3 V_pp_ and a frequency difference of 30 kHz while the frequency is swept from 110 MHz to 120 MHz at a frequency step of 1 MHz onto the source and gate electrodes respectively, and the drain current is imported to the input port of a lock-in amplifier (Signal Recovery 7265 DSP). The two AC signals are also imported to a homemade mixer circuit to pick up the signal at a frequency of 30 kHz, which is transmitted to the lock-in amplifier as a reference. SEM images of cells are taken by using FEI Quanta 200.

## Additional Information

**How to cite this article:** Zong, X. and Zhu, R. Zinc oxide nanorod field effect transistor for long-time cellular force measurement. *Sci. Rep.* **7**, 43661; doi: 10.1038/srep43661 (2017).

**Publisher's note:** Springer Nature remains neutral with regard to jurisdictional claims in published maps and institutional affiliations.

## Supplementary Material

Supplementary Information
