# Supplementary material for: Zinc oxide nanorod field effect transistor for long-time cellular force measurement
Source: Sci Rep. 2017 Mar 8;7:43661. doi: 10.1038/srep43661 (PMC5341559; doi:10.1038/srep43661)
Supplement: Supplementary Information [file srep43661-s1.doc]

**Supplementary information**

**Title: Zinc oxide nanorod field effect transistor for long-time cellular force measurement**

**Authors**: Xianli Zong and Rong Zhu*

State Key Laboratory of Precision Measurement Technology and Instruments, Department of Precision Instrument, Tsinghua University, Beijing 100084, China

**Figure S1. I-V Characteristics of the ZnO nanorods FET chip.** Measurement of currents by sweeping the voltage applied between source and drain from -3V to 3V for three times.

**Figure S2. Typical FET characteristics of the force sensor under a force of 5 nN.** Detected drain current (*ω*_1_=62 MHz, ∆*ω*=30 kHz) against peak-to-peak values of *δV_g_* and *δV_sd_*.

**Figure S3. The 14-hours stability test conducted by using the force sensor in a cell culture incubator at 37°C with 5% CO_2_ atmosphere.** 500uL DMEM supplemented with 10% fetal bovine serum is added in the pool on the micro-chip. AC sine-waves at certain frequencies, 0.3 V_pp_ with frequency difference of 30 kHz are applied onto the source and gate electrodes, respectively.

**Figure S4. Reproducibility of measurement.** (a) I-V characteristics of three different force sensors. (b) Measurements of cellular adhesion processes for 8 hours using three different sensors. AC sine-waves of 112MHz, 0.3 V_pp_ with frequency difference of 30 kHz are applied onto the source and gate electrodes, respectively.
